# Electrification of Heating—Requirements for Successful Wide‐Scale Deployment

**DOI:** 10.1002/wene.542

**Published:** 2024-12-17

**Authors:** Neil James Hewitt

**Affiliations:** ^1^ Ulster University Belfast UK

**Keywords:** electrification, heat pumps, integration

## Abstract

Electrification is potentially the most efficient method of decarbonization of space, water, and in certain instances, process heating through the deployment of electrically driven heat pumps. However, challenges are noted in terms of electricity network capacity that ultimately must influence a holistic approach to building/process heating demand reductions which in turn must influence heat pump development, heat pump operations, heat pump capital cost, and the role of thermal storage. Approaches to these challenges are presented from a global and, a UK and Ireland perspective, as for the UK and Ireland, it is often muted that the electricity network is less well suited to addressing the electrification of heating. Thus, this work will consider the likely cost‐effective heat demand reductions in buildings and processes, how might heat pumps operate in future electricity markets and systems, how can we make heat pumps cheaper to purchase and cheaper to operate and what is the role and type of energy storage in terms of demand side management. Thermal energy storage will be considered for space heating (30°C–80°C), water heating (60°C+ to negate legionella), and lower temperature industrial process (up to e.g., 300°C). Furthermore, an analysis of a UK housing development is presented as a retrofit case study for social housing, illustrating the impacts of the electricity network, and approaches that can be taken, for example, thermal storage, to minimize such impacts. Higher temperature systems will be considered for process applications.

## Graphical abstract

Heat pump test facilities at Ulster University for high‐temperature applications.

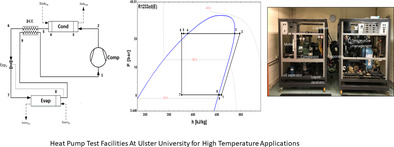

## 1 Introduction

The objective of this work is to demonstrate the potential of electrification of heating with vapor compression heat pumps, particularly in the UK and Ireland, through comparing global experiences with heat pumps in process and space heating and presenting an understanding of what needs to be carried out in the UK and Ireland for such positive global experiences to applied in the UK and Ireland. The potential for heat pumps in domestic space heating (which is well established outside the UK and Ireland) will be addressed. This work will also consider industrial heat pumps, (an established technology outside the UK and Ireland at lower temperatures, i.e., less than 100°C), but an emerging global technology at temperatures greater than 100°C. However, why address the electrification of heat?

Decarbonisation of heat is a global challenge. The International Energy Agency reported in 2021 that direct emissions from heating buildings grew by 5.5%, reaching a new high of 2500 Mt. CO_2_, 80% of direct CO_2_ emissions in the buildings sector with 50% of global energy used was heat and that heat usage represented 40% of global carbon dioxide emissions. Furthermore, 50% of the global heat used was for industrial processes with most of the remainder being for building and water heating. The use of electricity for space, water, and certain industrial heating process is not the only solution and infrastructure and end use may dictate alternative or hybrid solutions with, for example, hydrogen and biofuels. Nevertheless, electrification provides one of the most efficient methods of decarburization of space, water, and process heating when compared to hydrogen for example. Electricity (e.g., wind or solar) typically suffers 8% transmission losses and this applies to both cases. A wind farm or photovoltaic (PV) farm supplying electricity to an air‐source heat pump with a coefficient of performance of, for example, 2.45 would then see a system efficiency of 2.25. A hydrogen boiler at 90% efficiency is fed by an electrolyser at 70% efficiency, for example, would see a system efficiency of 58% (i.e., 0.58 in system efficiency terms), regardless of any safety concerns. Therefore, in terms of efficiency, electrification must be considered first. However, there are challenges in taking a purely efficiency‐based approach and these will now be discussed.

**FIGURE 1:**
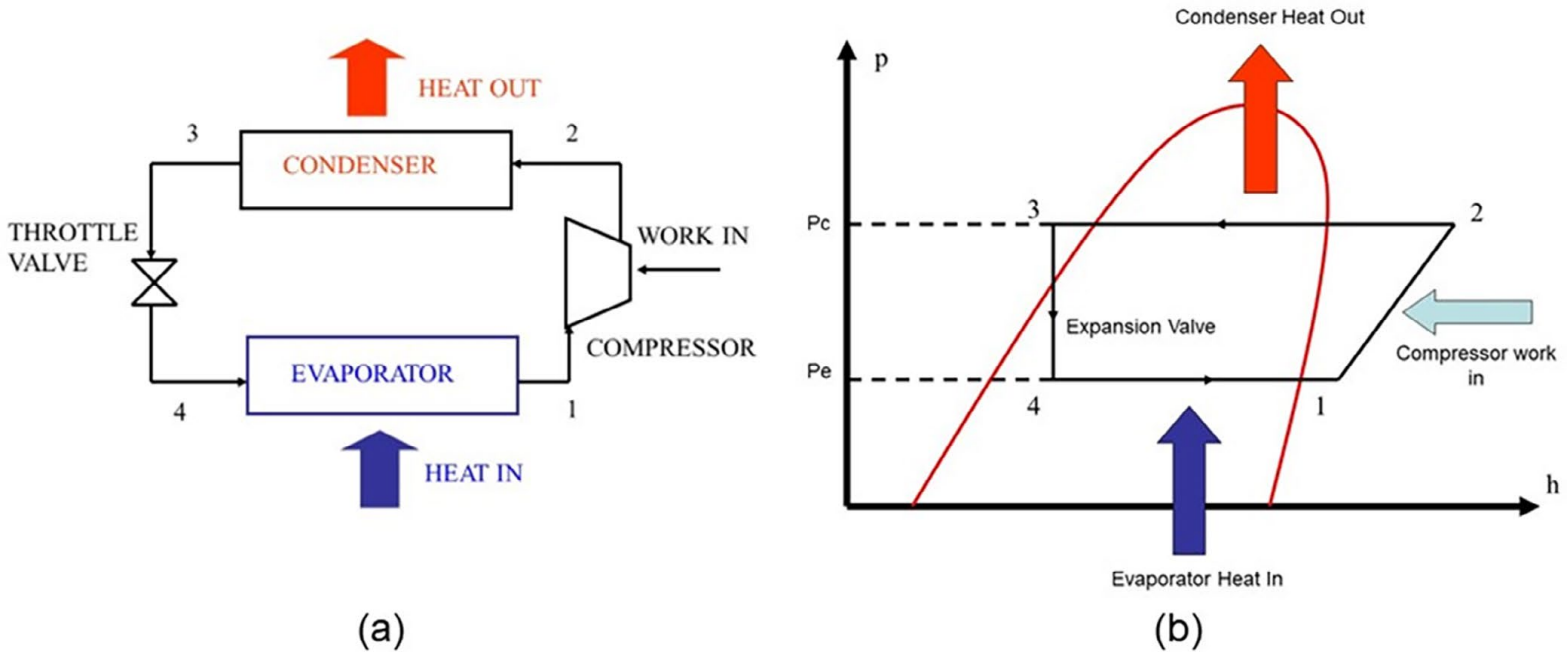
Heat pump schematic (a) and its representation via a pressure‐enthalpy chart (b).

Vapor compression heat pumps are typically electrically driven and the direct relationship between our most likely renewable energy resources (namely wind and photovoltaics) is the most efficient. However, electrification of heat can be direct or indirect resistance heating, heat batteries, and so on, but the heat pump is of interest here. Direct or indirect resistance heating has a coefficient of performance (COP) of 1, that is, 1 kW of electricity will produce (ideally) 1 kW of heat. A heat pump (Figure 1) takes a working fluid (refrigerant), boils it in an evaporator (process 4–1) at a temperature below that of the heat source (e.g., outside air) and a compressor raises the resulting vapor to a pressure, but more importantly a temperature higher than the space or process that requires heating (process 1–2). The heat is then delivered through condensation (process 2–3), leaving a high‐temperature liquid. The reverse of compression could be applied (expansion via a turbine) but it is more cost‐effective to expand the liquid to the lower evaporator pressure via an expansion valve (an orifice, capillary tube, or throttle valve, process 3–4). This type of expansion (pressure drop) results in some vapor being formed, but most of the working fluid is a cooler lower pressure liquid, ready to absorb heat again through evaporation. The efficiency increases are explained by the First Law of Thermodynamics in that where an electric resistance heater with convert 10 kW of electricity into 10 kW of heat, a vapor compression heat pump will extract 7.5 kW of heat from the air for example through evaporation of the working fluid, and through compressing the resultant gas with 2.5 kW of electricity to drive the compressor, the heat pump can deliver 10 kW of heat, that is, the sum of the heat and the electricity inputs.

With space and process heating traditionally delivered by fossil fuels, transitioning to the electrification of heating at any scale often becomes dependent upon electricity network capacity, and not just available renewable electricity resources. Thus, local existing electricity distribution networks (i.e., electricity cable capacity (feeders) and transformer capacity) are important. Ideally, new build should be considering the fossil fuel boiler phase‐out indicated by several countries, including the UK (2025) and Germany (where every newly installed heating system will have to be powered by 65% renewable energy by January 1, 2024). Consequently, efficiency of heat pumps (i.e., coefficient of performance, COP, and seasonal coefficient of performance SCOP) is paramount, where COP is defined as heat out divided by the electrical energy used and is an instantaneous measurement, for example, kW, whereas seasonal COP is measured across a year, and is thus measured in kWh. To reduce the electrical energy required for a heat pump, increasing, and decreasing the heat supply temperature and the heat delivery temperature respectively, with increased performance being achieved with the lower temperature lifts between heat supply and heat demand. Therefore, higher temperature heat sources, for example, waste heat, geothermal heat, and so on, and lower temperature heat demands, for example, large area hydronic radiators, underfloor heating, and optimized industrial heating processes, typically offer better performance in space and process heating applications, when compared to winter air temperatures as a heat source. However, air‐source heat pumps are considerably cheaper that ground‐source units, and the use of waste heat, geothermal heat, and so on, requires a heat network, for example, lower temperature 5th Generation heat networks, outlined by Gong et al. (2023), Davies et al. (Datacentres, 2016), Davies et al. (Underground railways, 2019), and such heat use in the community Revesz et al. 2022). Temperatures of 20°C or more are recoverable from the cooling of underground railways, data centers, and so on, and such temperature increase heat pump performance by 50% or more. Utilizing the 1st Law of Thermodynamics of Equation (1), the increase in Coefficient of Performance (COP) for a desired heating load (Q kW) will see a reduction in the electrical work required (W kW), this lowering the impact on existing electricity distribution networks.

(1) $$\mathrm{COP}=\frac{Q}{W}$$

## 2 Heat Pumps and Electrification of Heating

The global heat pump market has been growing (IEA “Heat Pumps,” 2024) and this is because of a combination of factors in countries that include financial supports to reduce capital costs, heat pump tariffs to improve operating costs and to reward demand side management, increasing taxes on fossil fuels, and bans on fossil fuel heating. However, there has been a recent reduction in heat pump sales as countries begin to withdraw subsidies for heat pumps.

**FIGURE 2:**
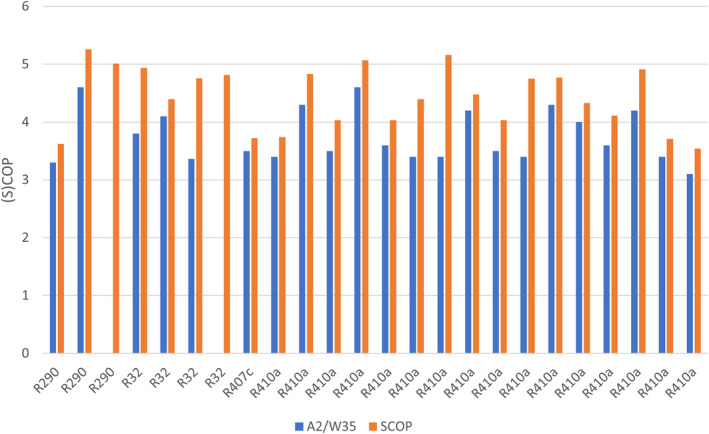
Manufactured domestic heat pump comparisons at EN14511 test standards (with each working fluid represented at two test conditions) (https://www.ost.ch/en/research‐and‐consulting‐services/technology/system‐technology/ies‐institute‐for‐energy‐systems/heat‐pump‐test‐center‐wpz).

Subsidies or indeed a lack of subsidies do not address heat pump impacts on existing electricity infrastructure. UK electrification of heating impacts are noted by Zhang et al. (2022) and Charitopoulos et al. (2023). So why is the local electricity distribution network deemed to be a challenge in the UK and Ireland? Afkousi‐Paqaleh et al. (2021) modeled UK Sweden, Germany, and France, for example, often have existing three‐phase electricity supplies in each home. This means that three‐phase power connections can use lower current (amps) for the same power. Heat pump performance, that is, reduction in electricity demand is also impacted internally through system design or externally through process/building design (to optimize/reduce demand temperature) and optimize/increase the supply temperature to the heat pump. Heat pumps utilize a working fluid that a given pressure, evaporates at a temperature below that of supply. This gas is compressed to a pressure, but more importantly temperature, above that of the demand temperature so that heat can be delivered to the building/process. Such fundamentals are important as the current generation of Fluorinated gases (F‐gases) (the most common working fluids) are an environmental concern. F‐gases in Europe are to be phased down to 25% of their 2015 supply volumes by 2024–2026 and to 5% by 2030–2032. A review of a sample of domestic heat pumps circa 35°C (Figure 2, derived from Heat Pump Test Centre WPZ Buchs online data) reveals the impacts of the pending phase of out of (higher Global Warming Potential, GWP). Figure 2 demonstrates that, for example, R290 (Propane) with a GWP of 3 (and thus well below F‐gas thresholds) still has a range of performance and while the other working fluids in use are likely to be phased down/out. Why is there such a range of performance? To increase performance, there are several approaches that include examining the temperature differences between the working fluid and the heat supply. Furthermore, reducing the temperature of the heat demand and increasing the temperature of temperature supply can be considered. Heat exchanger design is ultimately driven by the relationship between the heat exchanger heat transfer coefficient, area of the heat exchanger, and the (log mean) temperature difference heat supply and demand and the working fluid.

This is further complicated by the future trend towards natural working fluids. Carbon dioxide (CO_2_, R744) with introduce higher pressures and consequently, more materials and cost, while hydrocarbons, for example, Propane (C₃H₈, R290), iso‐butane (C_4_H_10_, R600a), and so on, introduce higher levels of risk with regards to flammability. Commercial systems and industrial higher temperature systems introduce ammonia (NH_3_, R717) and concerns over toxicity, water (H_2_O, R718), pentane (C_5_H_12_, R601), and so on with technical risk and flammability concerns. Such design considerations tend towards minimizing working fluid charge, but the mass flow of working fluid is directly proportional to the heat delivered by a heat pump (Box 1).

BOX 1 **Heat pump markets.**

Across Europe, nearly 3 million heat pumps were sold in 2022, an increase of almost 40% compared with the previous year. This is against a housing stock of nearly 200 million units and a previous cumulative heat pump installs of over 20 million heat pumps.

The following sections will address domestic, commercial, and industrial retrofit challenges, especially on the electricity network, and understand the implications of decarbonization of space and process heating via the electrification of heating through vapor compression heat pumps.

## 3 Vapor Compressions Heat Pumps for Space and Process Heating

The potential changes in working fluids have been noted and the types of heat pumps available will now be simplified into three basic styles: Air‐source, Ground‐source, and Water/Waste Heat/Heat Network Source. Again, in simple terms, air‐source heat pumps are typically the least expensive, and the expense increases with increasing heat source infrastructure, for example, horizontal or vertical heat loops for shallow ground source, boreholes for deeper ground source, and heat networks. Finally, as a simplification, the COP increases typically with each of these applications, for example, waste heat/heat networks are typically the most consistently warmest heat sources, ground source are less warm, and air is (in winter conditions) the least warm heat source. Thus, capital cost must be weighed against running costs for the heat pump element of the system whose lifetime is more than 10 years. For new build space heating and process heating applications, the change from fossil fuels to electrification of heating can be added to the infrastructure, but in retrofit (a very likely approach), assessments must be made of the existing electricity infrastructure to facilitate electrically driven vapor compression heat pump deployments.

### 3.1 Space Heating Heat Pumps

The typical range of space heating in a building range from a maximum of approximately 80°C to a minimum below 40°C. Options for air‐source, ground source, and water/district heating source will be presented.

#### 3.1.1 Domestic/Household Heat Pumps

**FIGURE 3:**
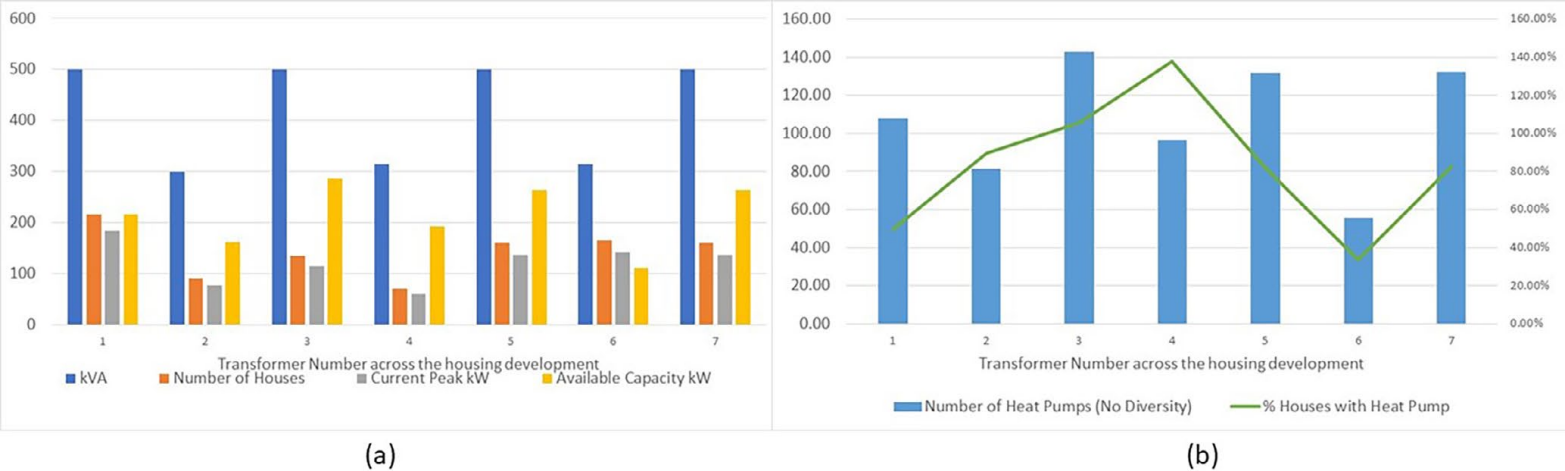
Summary of a local social housing development electricity network demands (a) and numbers of heat pumps that can be accommodated (b).

Using a local social housing development of 1000 houses with a typical peak heating demand of 5 kW, Figure 3 illustrates for the 7 transformers (ranging from 500 to 300 kVA), the likely available headroom on each transformer for an air source heat pump installation, assuming a COP of 2.5.

**FIGURE 4:**
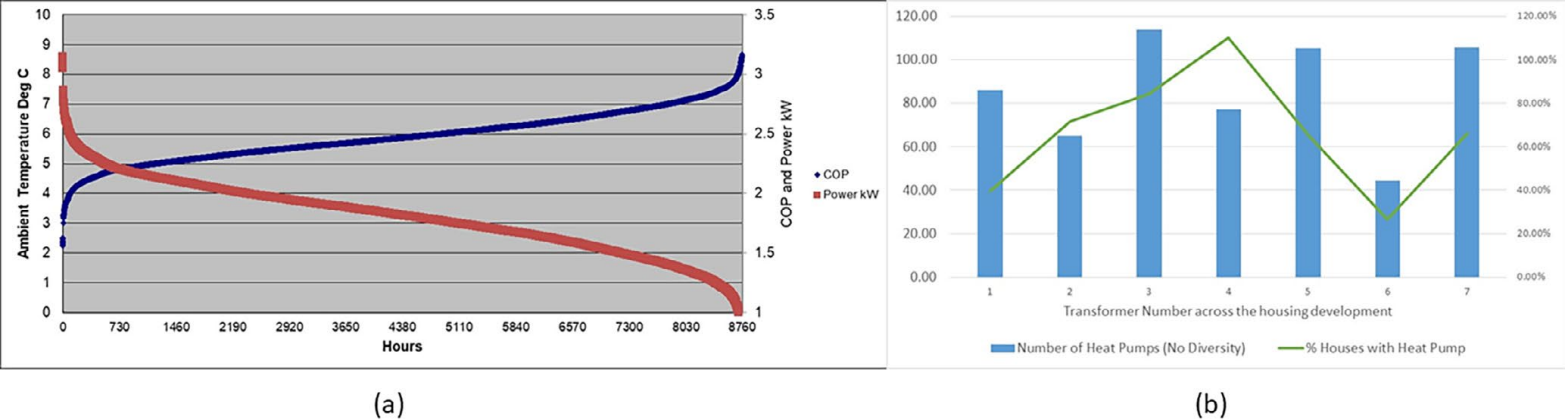
(a) Typical air temperatures and ASHP COP and (b) subsequent impacts on existing transformer infrastructure.

Assuming an average COP of 2.5 for an air‐source heat pump, and a diversity factor of 1.0 (diversity factor is limited for heat pumps in colder weather as all houses want to be heated at the same time), the different transformers can accommodate the maximum numbers of heat pumps noted in Figure 3. Transformers “3” and “4” have excess capacity and are not currently a challenge, although the roll‐out of electric vehicles is expected, and the impact on the low‐voltage distribution network may be significant (McGarry et al. 2023). However, air source heat pump performance deteriorates with colder air temperatures (Figure 4a), and while this may only happen for 2 weeks of the year, for example, the so‐called “Beast from the East” in 2018), the COP of an air source heat pump can be below 2.0 (Vorushylo et al. 2018). Under such conditions, all but Transformer 4 can deliver 100% heating by electrification via air source heat pumps.

Therefore, our options might include upgrading transformers and/or considering, for example, ground source heat pumps. Changing to a ground source heat pump with a COP of 4.0 (e.g., Miara, 2010), effectively makes only Transformer “1” and “6” with the inability to supply all houses with heat pumps.

The role of thermal storage is now interesting. If we were to supply 2 h of thermal storage, that is, 10 kWh of heat, and given the peak demand for space heating is between 4 and 8 p.m. in winter, and using Transformer “1” as an example, approximately 50% of the heat pump running times must not occur during the peak evening period. The role of thermal storage, that is, peak thermal demand of 5 kW per hour over 4 h at 50% of the time between 4 pm and 8 pm would lead to a maximum size of thermal storage of 10 kWh. This would be charged by the heat pump during off‐peak and/or night electricity supplies. The consequent is an increased capacity cost per household but being less expensive that ground source heat pumps. Space must be found for the storage cylinder, which would equate to over 250 L of water or lesser amounts of phase change material, for example, paraffin waxes (Nair et al. 2024). Alternatively, transformer upgrades (in the range of £250,000 per transformer, Western Power Distribution, 2022), would add a considerable cost to the £5000 for an air‐source heat pump per home and over £10,000 per home for a shallow ground source heat pump (Transformer Upgrade Costs, n.d.).

#### 3.1.2 Commercial Heat Pumps

Commercial heat pumps for space heating applications are essentially larger versions of domestic units and address similar space heating regimes. An area for decarbonization is our leisure facilities that include building complexes consisting of swimming pools, sports halls gyms, and so on and their accompanying changing and showering (domestic hot water) facilities. Assuming approximately 70,000 kWh of natural gas is used to heat a typical 25 m swimming pool, with 12 h a day heating, an insulated pool cover installed when not in use, this demand and that of the other leisure facilities would amount to approximately 200 kW peak thermal demands to be met by a heat pump. For the 15 leisure facilities identified in this region, all have the electrical capacity on their existing connection to accommodate a heat pump for swimming pool and other operations but running costs for an air‐source heat pump could be 8% higher than natural gas, while a ground source heat pump is 30% lower.

**FIGURE 5:**
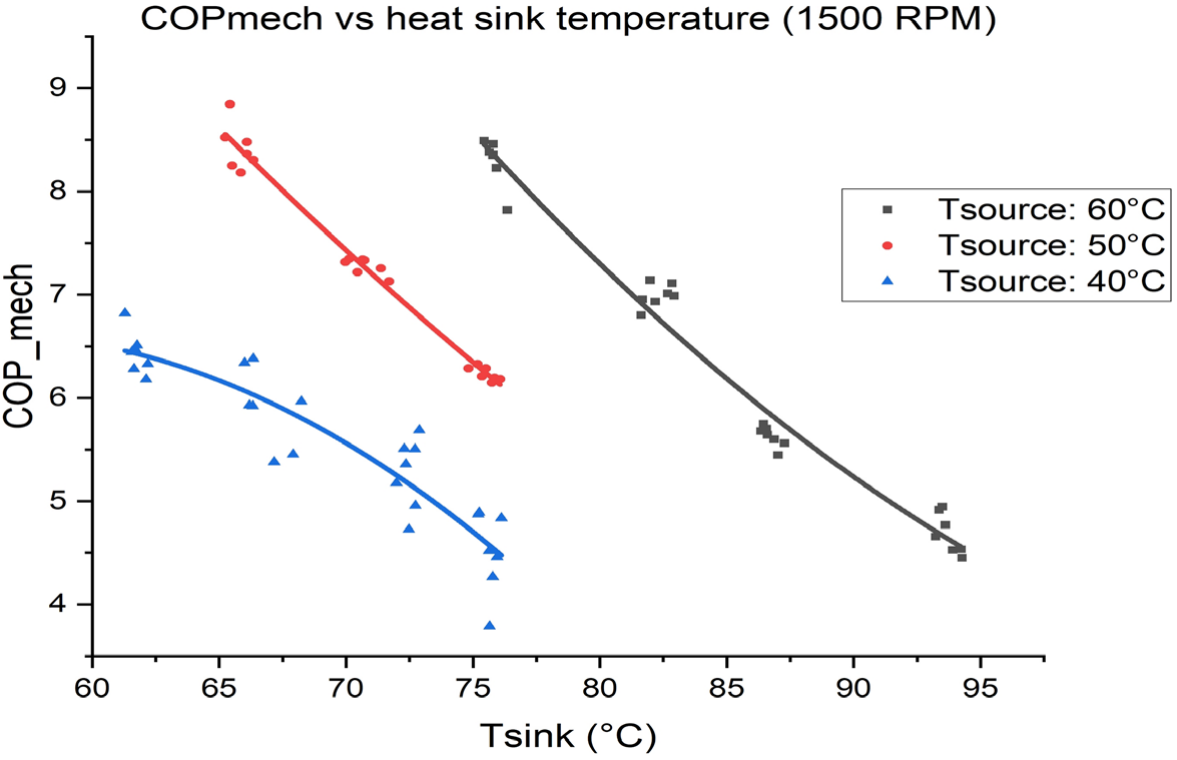
Ulster university experimental high temperature heat pump performance.

The role of district heating and larger applications as baseload customers is of interest, however. Experimental work at Ulster University reveals that a COP of such systems approaches 9.0, using R1233zd(E) and depending on the conditions encountered (i.e., temperature of heat supplied, and temperature required by the heating system of the building) (Figure 5). Tsource represents the district heating network temperature and Tsink represents the space heating or process heating need temperature.

### 3.2 Industrial Heat Pumps

Adamson et al. (2022) provided a global perspective for high‐temperature heat pumps with industrial process heat using 19% of final energy demand (which is equivalent to over 22,000 TWh) and 27% of this heat being in the range 100°C–200°C and 53% at temperatures above 500°C. The latter has often been considered territory beyond the reach of heat pumps and may become the domain of green hydrogen or other sources.

**FIGURE 6:**
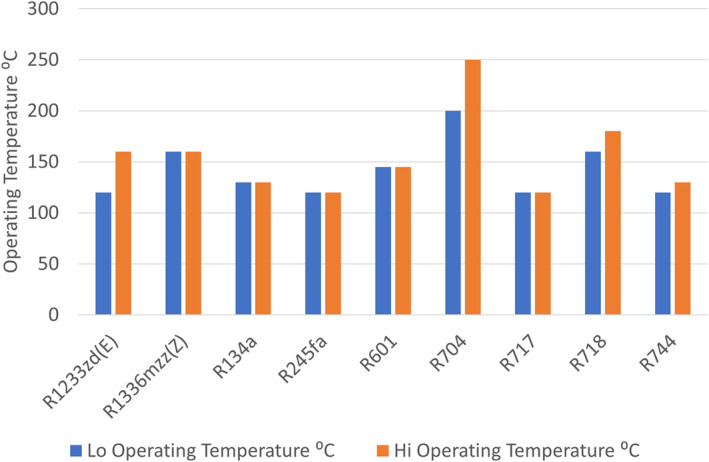
Current and proposed very high temperature heat pumps.

High supply temperatures are typically associated with large temperature lifts and therefore reduced thermodynamic performance. Increased system complexity has been noted, for example, intercoolers to optimize performance, while the components are subject to more challenging operating conditions. The limited operating performances and high investment costs of some of the currently available equipment inevitably results in limited economic performance (Arpagaus 2023). In addition, various synthetic refrigerants that might be used will be potentially phased out by stricter regulations concerning their environmental impact. Indeed, the use of R1233zd(E), is being questioned. Moving beyond F‐Gas regulations to the future phase out of working fluids proposed by the European Chemicals Agency, perfluoroalkyls (of which all but 5 refrigerants currently permitted are of this type) will be cut, leaving the space heating heat pump sector with, for example, R32 and R290 as their main choices. The US EPA has also banned certain working fluids, leaving R600, R717, and R744 and other natural fluids the remaining options. Figure 6 illustrates that from over 40 heat pump systems capable of delivering high temperatures, significant number may have to rethink their working fluids, for example, R1233zd(E), R1336mzz(Z), R134a, and R245fa (IEA HPT Annex 58, 2023) (https://heatpumpingtechnologies.org/annex58/).

And what of higher temperatures with natural working fluids. In developing new systems with global potential, the role of water as a refrigerant (R718) for heat pumps is increasingly relevant. The review of high‐temperature heat pumps utilizing R718 carried out by the International Energy Agency (IEA HPT Annex 58) demonstrates R718 heat pumps operating at up to 280°C for (semi‐open) mechanical vapor recompression systems, that is, the compressor discharge steam being utilized for an industrial process. Such compressors must address a high compressor discharge temperature, generated by R718's low molecular weight, high heat capacity ratio (Cp/Cv), and large specific volume. Furthermore, corrosion, lubricants, and related compression sealing (isentropic efficiency) are challenges.

R718 compressors tend to be physically large and practical systems tend to be in the MW thermal scale, as identified by the ongoing IEA HPT Annex 58. The main types of steam compressors currently include piston (40%–80% efficiency) (Klute et al. 2024), centrifugal (58%–79% efficiency), Roots (30%–67% efficiency), and screw compressors (50%–83% efficiency) (Ma et al. 2024) with oil free concepts resulting in the lower isentropic efficiencies. Given the costs noted by Zuhlsdorf (2023), a focus should be on that of extending the capabilities of existing equipment.

For R718, high Coefficients of Performance (COP ≥ 5) are possible (Šarevski and Šarevski 2017) and lower temperature experimental results for turbo (centrifugal) compressors are good but challenges remain in terms of increased impeller size with increased temperature lift, choice of bearings (gas‐lubricated, magnetic, etc.), efficiency, compactness, and the higher speeds and temperatures proposed to overcome compressor inefficiencies. Speeds of 100,000 to 650,000 rpm are now feasible at lower temperatures. Gas bearings will be initially sized for a rotor speed and impeller tip diameter for isentropic efficiency optimization. Rotor speed will affect bearing size. The speed of the electric motor will allow the diameter of the turbine to be calculated. Using a standard turbine layout with two journal bearings as a starting point, a steel alloy (e.g., AlMnMg) will lead to a lightweight impeller. Bearing clearance will dictate stability at high speeds leading to a requirement for optimal geometrical design and performance characteristics of the compressor. The impacts of peripheral speed, rotational speed, and impeller diameter on heat pump capacity for various evaporating and condensing temperatures will need to be evaluated as speed in the order of magnitude of Mach numbers will be required for high efficiency. The challenge is that of high impeller speeds of more than 100,000 rpm.

The future may see gases such as Argon (R740) operating at 500°C and above, if COPs and electricity networks can support such technology. In a traditional Brayton Cycle (e.g., a Combined Cycle Gas Turbine), thermal power is used to control, that is, less gas leads to less electricity and so forth. In theory, a Reversed Brayton Cycle cools a gas (i.e., gives up the heat to the heat sink). Thus, the gas volume will be reduced and technically the expander is smaller than the compressor. With less gas cooling, that is, a lesser demand for heat, the turbine will reduce speed. The controlling parameters are the pressure ratio and heat flows of both heat transfer processes as the control attributes. Thus, it may be appropriate to incorporate thermal storage at the higher and lower temperatures to manage heat flows for both process dynamics and demand side management applications. The gases for a Reversed Brayton Cycle include nitrogen, carbon dioxide, argon, and air with argon providing the best performance (Mahdi et al. 2022). As with Pimm, Cockerill, and Gale (2023), Argon was the fluid of choice due to its superior performance. A COP of 1.4 has been modeled lifting from 100°C to 500°C assuming compressor isentropic efficiencies of 0.8 and a turbine efficiency of 0.9 modeled in REFPROP v.10. A future proposed system would start with thermal control for compressor and turbine speed.

## 4 Conclusion

Heat pumps are once again going through a period of change. Since the 1980's, with the demise of Chlorofluorocarbons (Ozone Layer—CFCs), HCFC's in the 1990s, HFC's due to Global Warming Potential and now the Hydrofluoro‐olefins (HFO's) (the so‐called “forever chemicals”), while certain working fluids will remain, we will see the rise of natural working fluids, for example, Carbon Dioxide (R744), hydrocarbons, for example, Propane (C₃H₈, R290), iso‐butane (C_4_H_10_, R600a), and ammonia (NH_3_, R717). While this presents several not insurmountable technical challenges, it comes at a time when heat pumps where rapidly making inroads into the domestic sector heating market as well as beginning to have greater opportunities in the large‐scale heating and higher temperature process heating markets. It is no about the time granted for the industry to change over. The natural fluids are proven refrigerants with well‐defined properties and years of development behind them, so it becomes a matter of legislation (working fluids choice and health and safety), end‐user acceptability and overall performance, that is, not sacrificing the performance gains made with its subsequent increased in energy use and infrastructure demands. Nevertheless, heat pump performance must improve to manage integration with our existing electricity network, but in parallel, their capital cost must reduce. Finally, however, heat pumps have an important role in the decarbonization of heat. In displacing natural gas for space heating in the UK, an air‐source heat pump achieving a COP of 2.5 will save over 2 t of CO_2_ emissions when compared to oil‐fired heating and over 1 t of CO_2_ emissions when compared to gas‐fired heating. A (ground‐source) heat pump with a COP of 4 will save 2.6 t of CO_2_ emissions when compared to oil‐fired heating and 1.6 t of CO_2_ emissions when compared to gas‐fired heating. These savings assume 100% renewable electricity, which appears to one of the easier of our decarbonization pathways. And finally, for very high temperature, a new horizon beckons and initially, engineering limitations must be overcome to understand if practical solutions will arise.

## Author Contributions

**Neil James Hewitt:** funding acquisition (equal), investigation (equal).

## Conflicts of Interest

The author declares no conflicts of interest.

## Related WIREs Articles

Technological learning: Lessons learned on energy technologies

## Data Availability

Data sharing is not applicable to this article as no new data were created or analyzed in this study.
